# 肝细胞生长因子诱导敏感非小细胞肺癌细胞对吉非替尼耐药及机制的研究

**DOI:** 10.3779/j.issn.1009-3419.2013.01.01

**Published:** 2013-01-20

**Authors:** 香兰 玄, 昌善 安, 彩存 周

**Affiliations:** 1 133000 延吉，延边大学附属医院呼吸内科 Department of Respiratory Disease, Yanbian University Hospital, Yanji 133000, China; 2 200433 上海，上海市肺科医院肿瘤科 Department of Oncology, Tongji University Affiliated Shanghai Pulmonary Hospital, Shanghai 200433, China

**Keywords:** 肝细胞生长因子, 吉非替尼, c-Met, 耐药, 肺肿瘤, HGF, Gefitinib, c-Met, Resistance, Lung neoplasms

## Abstract

**背景与目的:**

肝细胞生长因子（hepatocyte growth factor, HGF）受体（c-Met）可能与非小细胞肺癌（non-small cell lung cancer, NSCLC）对吉非替尼耐药有关。本研究旨在探讨HGF诱导不同基因型NSCLC对吉非替尼耐药及耐药机制。

**方法:**

选择NSCLC细胞*EGFR*突变型PC-9和*EGFR*野生型H292，用HGF诱导这两株细胞，通过MTT法检测细胞增殖，PI法检测细胞周期，Annexin V-PE法检测细胞凋亡，应用免疫印迹（Western blot）技术检测细胞中c-Met、p-Met的表达。

**结果:**

吉非替尼对PC-9和H292的生长抑制作用呈浓度依赖性，HGF诱导后吉非替尼抑制两种细胞的生长曲线往右移。PC-9和H292的HGF和吉非替尼处理组（HG）比吉非替尼处理组（G）均有更高的存活率（*P* < 0.05），HG组与G组两组间细胞凋亡及细胞周期无统计学差异（*P* > 0.05）。HGF明显增加PC-9和H292中p-Met的表达。吉非替尼能明显抑制HGF诱导PC-9增加表达的p-Met，但不能抑制HGF诱导H292增加表达的p-Met。

**结论:**

HGF可诱导敏感肺癌细胞PC-9和H292对吉非替尼耐药，HGF刺激c-Met磷酸化可能是敏感肺癌细胞对吉非替尼耐药的重要机制。

## Abstract (en)

**Background and objective:**

Previous studies have reported that Met might be related to gefitinib resistance in non-small cell lung cancer (NSCLC). The present study aims to explore the mechanism of hepatocyte growth factor (HGF)-induced gefitinib resistance in different gene types of sensitive NSCLC *in vitro*.

**Methods:**

The PC-9 and H292 cell lines were chosen and induced by HGF. The cell survival was measured using MTT assay, the cell cycle distribution was measured using PI assay, and cell apoptosis with an Annexin V-PE assay, respectively. The c-Met and p-Met protein expression was determined via Western blot analysis.

**Results:**

Gefitinib inhibited the growth of PC-9 and H292 cells in a dose-dependent manner. The concentration-survival curves of both cell lines shifted to the right when induced with HGF. HGF did not affect PC-9 and H292 cell proliferation. The cell also had a higher cell survival rate when treated with HGF and gefitinib compared with that under gefitinib alone (*P* < 0.05). The apoptotic rate and cell cycle progression showed no significant difference between the HG and G group (*P* > 0.05). HGF stimulated Met phosphorylation in the PC-9 and H292 cells. Gefitinib inhibited the HGF-induced Met phosphorylation in PC-9 cells, but not in H292 cells.

**Conclusion:**

HGF induces gefitinib resistance in PC-9 and H292 cells. HGF-induced Met phosphorylation may be an important mechanism of gefitinib resistance in sensitive NSCLC.

吉非替尼是一种选择性表皮生长因子受体酪氨酸激酶抑制剂（epidermal growth factor receptor tyrosine kinase inhibitor, EGFR-TKI），是目前最常用的治疗非小细胞肺癌（non-small cell lung cancer, NSCLC）分子靶向药物。由于吉非替尼对患者的选择性，仅部分NSCLC患者对吉非替尼有效，存在原发或获得耐药，这种耐药机制不十分清楚。c-Met是肝细胞生长因子（hepatocyte growth factor, HGF）的受体，新近研究^[1]^显示，肺癌组织中*MET*基因的扩增与对吉非替尼获得性耐药有关。本研究选择NSCLC细胞*EGFR*突变型PC-9和*EGFR*野生型H292，用HGF诱导这两株细胞，应用MTT法检测细胞增殖、周期及凋亡，利用蛋白质免疫印迹技术检测细胞中c-Met、p-Met的表达，旨在研究HGF诱导不同基因型NSCLC对吉非替尼耐药及耐药机制。

## 1 材料与方法

### 1.1 材料

人肺癌细胞株PC-9、H292由上海肺科医院中心实验室冻存提供; HGF购自以色列PROSPEC公司，吉非替尼购自武义金色年华药物研发公司，Annexin V-PE Apoptosis Detection Kit为BioVision公司产品，Flow Cytometry Analysis of Cell Kit为上海杰美基因医药有限公司产品，鼠抗人p-EGFR（Tyr1068）、兔抗人p-Met（Tyr1234/1235）及c-Met购自Cell Signaling Technology公司，小鼠抗人β-actin单抗及辣根过氧化物酶标记的羊抗兔、羊抗小鼠IgG抗体为武汉博士德产品。

### 1.2 细胞培养及药物配制

将存有PC-9和H292的液氮冻存管迅速放入37 ℃恒温水浴箱快速融化，PBS洗涤后加入10 mL含10%胎牛血清的DMEM培养基，5%CO_2_、37 ℃恒温细胞培养箱中孵育，每3-4天换液传代。HGF 10 μg加入100 μL无菌双离子水稀释成100 μg/mL的母液。吉非替尼原料用DMSO溶解稀释成浓度为50 μmol/mL的母液，用药时DMSO终浓度要小于0.1%。

### 1.3 测定细胞活性及药物半数抑制浓度（IC_50_）

取100 μL含细胞数为5×10^3^个的细胞悬液接种于96孔板。待细胞贴壁后加入100 μL含有HGF或（和）不同浓度吉非替尼的培养液。72 h后每孔内加入20 μL MTT（5 mg/mL），孵育4 h，离心，弃上清液，每孔加入200 μL DMSO，摇床上充分混匀1 h，待结晶完全溶解。最后用酶标仪测量波长530 nm时OD值; 细胞存活率=（每组平均OD值－试剂空白/对照组平均OD值－试剂空白）×100%;实验重复3次，用细胞存活率做出量效曲线，用作图法分析得出两种药物对不同细胞的IC_50_。

### 1.4 细胞周期的检测

取1×10^5^个对数生长期细胞接种于6孔板，贴壁后弃原培养液，用PBS清洗2遍，加入含有HGF或（和）吉非替尼培养液，培养48 h，胰酶消化并收集全部细胞，离心去上清液，冷PBS洗涤2次，加1 mL固定液，固定2 h以上。离心去上清液，冷PBS洗涤2次, 加入500 μL标记液（500 μL Assay Buffer+10 μL RNase A+10 μL PI），避光、室温孵育30 min，过滤后流式细胞仪检测细胞周期，用Multicycler 3.0软件分析结果。实验重复3次。

### 1.5 细胞凋亡的检测

取1×10^5^个对数生长期细胞接种于6孔板，贴壁后弃原培养液，用PBS清洗2遍，加入含有HGF或（和）吉非替尼培养液，培养48 h和72 h，胰酶消化并收集全部细胞，离心去上清液，冷PBS洗涤2次。加入500 μL Binding Buffer悬浮细胞，加入5 μL Annexin V-PE混匀，避光、室温反应5 min，过滤后流式细胞仪检测细胞凋亡。实验重复3次。

### 1.6 Western blot检测细胞蛋白

取对数生长的细胞，给予相应处理后立即冰上裂解细胞，4 ℃、12, 000 rpm、30 min、离心收集各组蛋白裂解液。用BCA蛋白定量法蛋白定量。取30 μg-40 μg蛋白经8%-10%SDS-PAGE电泳分离后，转印至NC膜上，用5%脱脂奶粉封闭1 h，一抗孵育4 ℃过夜，TBST洗膜10 min、3次后二抗室温摇床孵育1 h，TBST洗膜5 min、5次后ECL化学发光试剂显色、曝光成像。

### 1.7 统计学分析

数值以均数±标准差来表示，采用SPSS 11.5软件进行统计学分析。以配对*t*检验进行统计学比较，*P* < 0.05为差异有统计学意义。

## 2 结果

### 2.1 吉非替尼或/和HGF处理的药物浓度-生长曲线

吉非替尼对PC-9和H292的IC_50_分别为（0.05±0.01）μmol/L和（0.17±0.05）μmol/L。随着吉非替尼浓度的升高，72 h内细胞存活率减少，即吉非替尼对PC-9和H292的生长抑制作用呈浓度依赖性，药物浓度越高细胞存活率越低（图 1）。当PC-9和H292用HGF（40 ng/mL）和不同浓度吉非替尼同时处理时，其药物浓度-生长曲线往右移（图 1）。当用HGF（40 ng/mL）和不同浓度吉非替尼同时处理时，吉非替尼对PC-9和H292的IC_50_值均大于1 μmol/L，而且估计H292的IC_50_远大于1 μmol/L。

**1:**
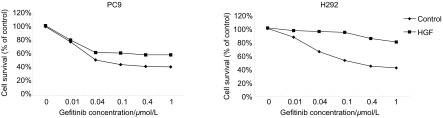
吉非替尼或/和HGF处理的浓度-存活率曲线（HGF诱导时曲线往右移） The concentration-survival curve when treating with gefitinib or/and HGF (curve shifts right when induced by HGF)

### 2.2 不同浓度HGF诱导PC-9和H292对吉非替尼耐药

实验共分4组：对照组（C）、HGF诱导组（H）、吉非替尼处理组（G）、HGF诱导和吉非替尼处理组（HG）。PC-9和H292先用HGF（40 ng/mL）诱导24 h后再用吉非替尼处理72 h，结果与G组存活率无统计学差异（*P* > 0.05），而同时用HGF和吉非替尼处理组与单纯用吉非替尼处理组细胞存活率更高，两组间有统计学差异（*P* < 0.05）（图 2）。所以上述药物浓度-存活率以及接下来的实验均采用HGF和吉非替尼同时加入的方法来处理。HGF（40 ng/mL）并没有影响肺癌细胞PC-9和H292的增殖，但HGF能诱导肺癌细胞对吉非替尼耐药，G组和HG组的存活率有统计学差异（*P* < 0.05）（图 3）。用低浓度HGF（20 ng/mL）处理没有影响PC-9和H292的增殖，但低浓度HGF也能诱导肺癌细胞对吉非替尼耐药，G组和HG组的存活率有统计学差异（*P* < 0.05）（图 3）。

**2:**
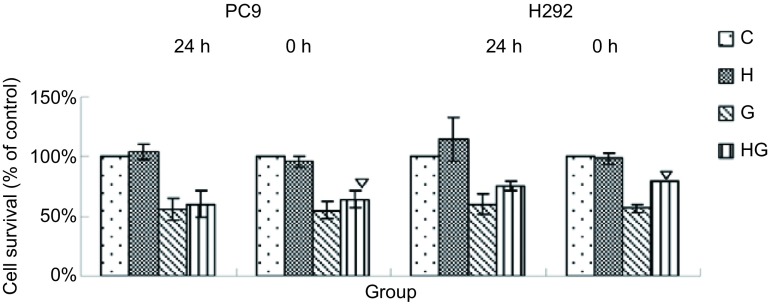
HGF（40 ng/mL）诱导不同时间（0 h、24 h）后吉非替尼（1 μmol/L）处理对肺癌细胞PC-9和H292存活率的影响。C：对照组; H：HGF诱导组; G：吉非替尼处理组; HG：HGF诱导和吉非替尼处理组。▽:与吉非替尼处理组（G）相比，*P* < 0.05。 The effects of cells survival when treating with gefitinib after induced by HG (40 ng/mL) in different time (0 h, 24 h). C: control group; H: HGF group; G: gefitinib group; HG: HGF+gefitinb group. ▽: compared with the G group, *P* < 0.05.

**3:**
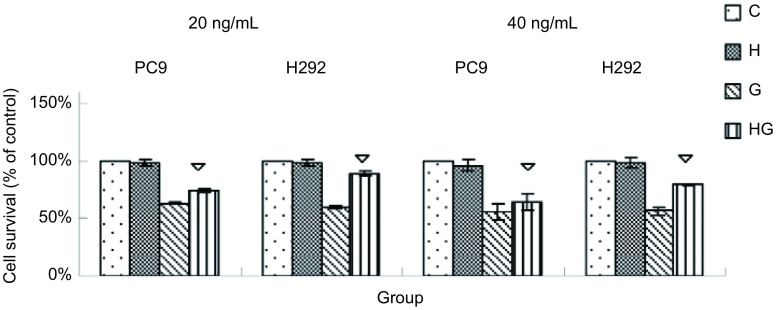
HGF（20 ng/mL、40 ng/mL）或/和吉非替尼对肺癌细胞存活率的影响。▽：与吉非替尼处理组（G）相比，*P* < 0.05。 The effects of cells survival when treated with gefitinib or/and HGF (20 ng/mL, 40 ng/mL). ▽: compared with the G group, *P* < 0.05.

### 2.3 HGF诱导吉非替尼耐药对细胞凋亡的影响

光镜下观察，吉非替尼能明显促进PC-9凋亡，而吉非替尼对促进H292的凋亡作用并不明显。因此我们只选择凋亡作用明显的PC-9来说明凋亡在HGF诱导吉非替尼耐药过程中的影响。根据MTT检测的阳性结果选择不同浓度HGF（20 ng/mL、40 ng/mL）及吉非替尼（0.1 μmol/L）不同时间（48 h、72 h）处理PC-9。结果HG组凋亡率与G组相比有减少趋势，但无统计学差异（*P* > 0.05）（图 4）。

**4:**
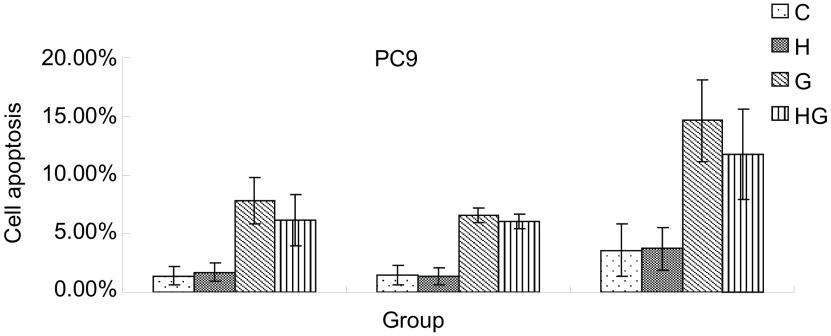
HGF诱导耐药对细胞凋亡的影响 The effects of apoptosis in gefitinib resistence induced by HGF

### 2.4 HGF诱导吉非替尼耐药对细胞周期的影响

PC-9和H292经HGF（20 ng/mL）或/和吉非替尼（0.1 μmol/L）各处理48 h。吉非替尼明显增加PC-9的G_1_期细胞比例，但G组与HG组比较无统计学差异（*P* > 0.05）。吉非替尼略增加H292的G_1_期细胞比例，但与HG组比较无统计学差异（*P* > 0.05）（图 5）。

**5:**
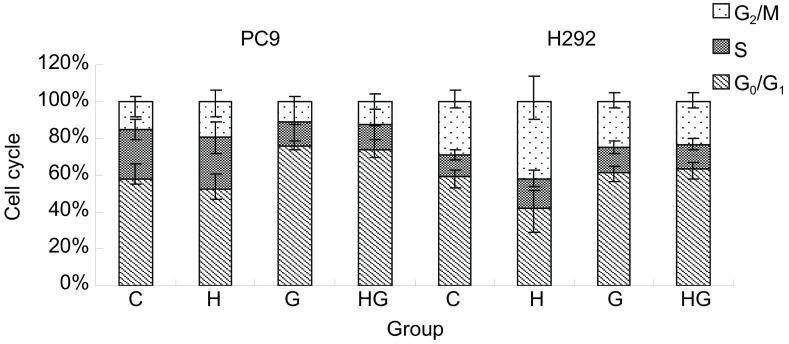
HGF诱导耐药对细胞周期的影响。G_0_/G_1_：DNA复制前期; S：DNA复制期; G_2_/M：DNA分裂期。 The effects of cell cycle in gefitinib residence induced by HGF. G_0_/G_1_: prophase of DNA; S: replicative phase of DNA; G_2_/M: mitotic phase of DNA.

### 2.5 HGF激活c-Met磷酸化

为了明确HGF诱导肺癌细胞对吉非替尼耐药机制，应用Western blot检测c-Met和p-Met表达。HGF作用能明显刺激PC-9和H292中c-Met的自身磷酸化（图 6）。PC-9为EGFR突变株，对吉非替尼十分敏感，吉非替尼对PC-9的IC_50_值为0.05 μmol/L。PC-9的HG组c-Met的磷酸化表达不明显，但与G组相比c-Met的磷酸化表达稍有增加（图 6）。H292为EGFR野生株，但对吉非替尼敏感，吉非替尼对H292的IC_50_值为0.17 μmol/L。HG组以及H组c-Met的磷酸化均明显增加，H292的HG组与G组相比c-Met的磷酸化明显增加（图 6）。

**6:**
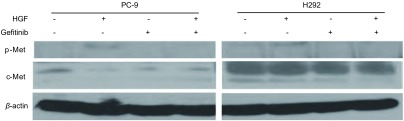
HGF诱导PC-9和H292细胞c-Met及p-Met表达 Expression of c-Met and p-Met in PC-9 and H292 induced by HGF

## 3 讨论

EGFR-TKIs对NSCLC患者有效率约为10%-20%^[2]^，也就是说80%-90%的NSCLC患者对EGFR-TKIs耐药。*EGFR*突变与EGFR-TKIs有效率有很大的相关性，但突变患者中70%-75%有效^[3]^，而25%~30%突变者则无效。NSCLC的野生型患者有10%~15%的有效率，野生型者中有85%-90%是耐药患者^[4]^。NSCLC患者存在原发性或获得性对EGFR-TKIs耐药，其耐药机理尚未完全明确，*EGFR*的二次突变与*MET*基因的扩增被认为是吉非替尼获得性耐药的重要机制。HGF属于成纤维细胞的衍生因子，恶性肿瘤患者血清中HGF含量明显升高，与肿瘤的侵袭状态密切相关^[5]^。HGF与特异性c-Met受体结合而发挥作用，促进多种组织细胞增生分裂、促细胞运动和促血管生成^[6]^。

我们选择对吉非替尼敏感的*EGFR*突变肺腺癌细胞型PC-9和*EGFR*野生型H292，这两株细胞不存在*EGFR*^T790M^突变或*MET*扩增或*KRAS*突变等吉非替尼耐药相关因素。本研究细胞活性检测显示HGF单独没有促进细胞增殖，但HGF诱导与非HGF诱导的药物浓度-存活率曲线相比，HGF诱导的药物浓度-存活率曲线明显往右移，提示HGF诱导细胞存活。PC-9和H292的HG组比G组均有更高的存活率（*P* < 0.05）。从IC_50_的变化、药物浓度-存活率曲线、细胞存活率的结果看出，HGF不仅诱导敏感肺癌细胞对吉非替尼耐药，而且与*EGFR*突变型和野生型无关。在HGF和吉非替尼用浓度相同处理下，耐药影响在*EGFR*野生型中的表现更明显，提示野生型细胞更容易受HGF的干扰。

本研究中HGF诱导的PC-9凋亡率并没有比对照组减少，可忽略细胞凋亡作用在HGF诱导耐药中的影响。HGF能明显减少H292的G_1_期细胞比例，增加S期和G_2_期细胞比例。经过吉非替尼处理的诱导组G_1_期细胞比例与非诱导组相比没有差异，提示细胞周期的影响可能与HGF诱导耐药无关。

肿瘤细胞除了表达EGFR外同时还表达其它含酪氨酸激酶活性的跨膜受体，称之为EGFR旁路TK信号，包括c-Met，它们所引导的信号通路常常重叠、功能上发生碰撞^[7]^。c-Met广泛存在于多种正常组织细胞和体内外恶性肿瘤细胞内。正常情况下HGF/c-Met信号途径参与胚胎发生，而异常的HGF/c-Met信号途径与肿瘤的发生和发展密切相关，特别是在促进肿瘤细胞的侵袭和转移方面起重要作用^[8]^。在NSCLC患者中c-Met阳性表达者比c-Met阴性表达者存活率低，HGF和c-Met共表达者比任何一种阳性表达者或者两种均阴性表达者相比存活率明显降低^[9]^。

本实验显示，HGF诱导组包括HG组和H组均增加表达c-Met的磷酸化。两种细胞的HG组均比G组增加表达c-Met的磷酸化，这可能是HGF诱导敏感细胞对吉非替尼耐药的机制。本研究中PC-9是EGFR突变株，HGF诱导的c-Met磷酸化被吉非替尼抑制; 而H292是EGFR野生株，HGF诱导的c-Met磷酸化不被吉非替尼抑制。虽然在*EGFR*突变株，HGF诱导的c-Met的磷酸化被吉非替尼所抑制，但从*EGFR*野生株的表现中可以看出，HGF通过激活c-Met的磷酸化诱导敏感肺癌细胞对吉非替尼耐药。EGFR和c-Met都拥有广泛的信号网络，两种受体介导的信号通路广泛交错，分别依赖于EGFR和c-Met的细胞的信号网络。吉非替尼抑制HGF活化的c-Met的磷酸化在不同基因类型细胞之间的表现不一致，这为临床指导治疗不同基因型耐药NSCLC提供理论依据。

HGF虽然是本研究中介入的外源性生长因子，但先前就有文献^[10]^报道肺癌组织能够高表达HGF。HGF以自分泌或旁分泌的方式作用于其受体c-Met^[11]^，HGF诱导NSCLC对吉非替尼耐药可能属于原发性耐药。本实验提示少数*EGFR*突变患者及多数*EGFR*野生型患者对吉非替尼耐药的可能原因是HGF诱导NSCLC细胞表达的c-Met发生磷酸化。HGF/c-Met系统介导NSCLC对吉非替尼的耐药，如果联合HGF/c-Met信号靶向治疗可能有利于提高肺癌患者对吉非替尼的敏感性。
